# Perinatal Anxiety and Depressive Symptoms and Maternal Parenting Behavior During the First Three Years Postpartum: A Systematic Review

**DOI:** 10.1155/da/1801371

**Published:** 2025-05-19

**Authors:** Ana Morais, Rita Pasion, Tiago Miguel Pinto, Giulia Ciuffo, Chiara Ionio, Raquel Costa, Inês Jongenelen, Diogo Lamela

**Affiliations:** ^1^Digital Human-Environment Interaction Labs (HEI-Lab), Lusófona University, Porto, Portugal; ^2^Faculty of Psychology, Catholic University of the Sacred Heart, Milan, Italy; ^3^EPIUnit – Institute of Public Health, University of Porto, Porto, Portugal; ^4^Laboratory for Integrative and Translational Research in Population Health (ITR), University of Porto, Porto, Portugal

**Keywords:** anxiety symptoms, comorbidity, depressive symptoms, parenting, perinatal, postpartum

## Abstract

**Background:** Perinatal anxiety and depressive symptoms are prevalent and may influence parenting behaviors, yet their effects across distinct parenting dimensions remain unclear. Despite frequent co-occurrence, their combined impact is underexplored. Additionally, variability in how parenting behaviors are conceptualized hinders synthesis across studies. Categorizing parenting behaviors into protection, control, and guided learning provides a structured framework for understanding these associations.

**Objectives:** This review aimed to assess (1) the differential associations between perinatal anxiety,depressive symptoms, and parenting behaviors across the protection, control, and guided learning dimensions, and (2) associations between comorbid anxiety-depressive symptoms and parenting behaviors.

**Methods:** A systematic search was conducted across four databases in January 2024. Studies were included if they assessed perinatal anxiety and depressive symptoms and their associations with parenting behaviors during the first 3 years postpartum. Parenting behaviors were categorized into protection, control, and guided learning, and risk of bias was systematically evaluated. This review is registered with PROSPERO (CRD42023337333).

**Results:** From 9673 screened documents, 20 studies met inclusion criteria. Associations were most frequent in the protection dimension, with higher perinatal anxiety and depressive symptoms linked to lower maternal sensitivity and responsiveness. In the control dimension, findings were mixed, with some studies linking maternal anxiety and depressive symptoms to greater controlling behaviors, while others found no significant associations. In the guided learning dimension, null findings predominated, though some studies identified links between higher anxiety, depressive symptom levels, increased intrusiveness, reduced cognitive stimulation, and disengagement. Few studies examined comorbid anxiety and depressive symptoms, but preliminary findings suggest associations with lower maternal sensitivity and reduced guided learning behaviors.

**Conclusions:** Despite heterogeneity across studies, protection-related parenting behaviors were most consistently associated with perinatal anxiety and depressive symptoms. These findings highlight the need for targeted assessments and interventions to support affected mothers and their children.

## 1. Introduction

The transition to parenthood is a challenging time for both parental adaptation and infant development [1, 2]. During this period, parents undergo significant biological, psychological, and interpersonal changes that require a reorganization of self-representations, emotional regulation, family roles, social networks, and work–life balance [1, 3, 4]. Early parenting behavior is a significant protective factor to the child's emotional, social, and cognitive development, as it establishes a secure base that supports exploration and autonomy [5, 6]. However, the complexity of this transition is further, compounded by increased vulnerability to mental health issues, particularly anxiety and depression, which can significantly affect parenting behavior [2, 7].

Parenting during the early years of a child's life can be conceptualized as a dynamic and co-constructed relationship in which parents and children mutually influence and adapt to each other's behaviors and developmental trajectories [8]. Despite over six decades of research aimed at defining and conceptualizing parenting, a lack of consensus persists regarding its core dimensions. Numerous theoretical frameworks have sought to capture the essential components, functions, and developmental progression of parenting, yet each is constrained by specific conceptual and methodological limitations [9, 10].

One of the most influential frameworks is Baumrind's typology of parenting styles, which classifies parenting along two key dimensions: responsiveness and demandingness [11–13]. Responsiveness encompasses warmth, support, and the encouragement of autonomy, while demandingness refers to behavioral control, supervision, and the establishment of expectations. These dimensions define four parenting styles: authoritative, authoritarian, permissive, and neglectful [12, 14]. While foundational, Baumrind's framework has been critiqued for its reliance on bipolar dimensions, which oversimplify parenting behaviors, and its static nature, which limits its applicability across developmental stages and diverse contexts.

The six dimensions of parenting model [15] extends this conceptualization by introducing six unipolar dimensions: warmth, rejection, structure, chaos, autonomy support, and coercion. By treating dimensions as independent, this model captures the complexity of parenting behaviors, allowing for the coexistence of traits, such as warmth and coercion. However, despite its greater flexibility, the model does not account for the dynamic nature of parenting tasks, which evolve in response to the developmental and contextual needs of children.

These limitations highlight the need for a developmentally sensitive framework that emphasizes the dynamic and adaptive nature of parenting behaviors, which have evolved to address specific needs tied to the child's age, developmental stage, and environmental context. A recent integrative model informed by evolutionary developmental psychology provides, such as approach, identifying three core dimensions: protection, control, and guided learning [16–18]. Grounded in an evolutionary-developmental perspective, this model assumes that parenting behaviors reflect adaptive strategies to meet the challenges of ensuring a child's survival, fostering socialization, and promoting skill acquisition across different developmental stages [16]. Importantly, these dimensions are dynamic and interdependent, adapting to the child's changing needs and contextual demands while operating synergistically to optimize developmental outcomes.

The protection dimension encompasses behaviors aimed at shielding the child from potential physical and psychological harm, creating a stable and nurturing environment, and being responsive to the child's emotional needs. Protective behaviors are critical in fostering a secure attachment, which serves as a foundation for healthy infants' psychological development [5, 16, 18, 19]. The control dimension involves the regulation of the child's behavior to align with family and societal norms. This dimension includes setting boundaries, monitoring activities, and enforcing rules and consequences [11, 16–18]. Control is not merely about imposing discipline but also about guiding the child towards self-regulation and appropriate social conduct. Effective control balances firmness with empathy, ensuring that disciplinary measures are perceived as fair and supportive [16–18, 20]. The guided learning dimension refers to parental strategies intended to facilitate the child's acquisition of knowledge, skills, and competencies. This dimension involves active engagement in the child's educational processes and emphasizes the importance of providing scaffolding learning experiences and fostering an environment that encourages the child's cognitive improvement [17, 18]. This three-dimensional model offers a structured and developmentally responsive framework for understanding how parental behaviors manifest and adapt across different contexts. Moreover, it underscores the interdependence of these dimensions, wherein effective guided learning often relies on the emotional security established through protection, while control provides the structure necessary for learning to occur.

Anxiety and depression are among the most prevalent mental health disorders during the perinatal period. Global estimates suggest that the prevalence of perinatal anxiety disorders is around 17.4% [21] and depression is approximately 26.3% [22]. Some research suggests that these disorders can be associated with suboptimal parenting behaviors [23]. For instance, depressive symptoms have been linked to reduced sensitivity, emotional unavailability, and inconsistent responses to infant's cues [24–26]. Similarly, perinatal anxiety is associated with hypervigilance, overprotectiveness, and excessive control, potentially limiting the child's autonomy, and fostering an environment of heightened emotional distress [27–29].

Despite these known effects, research on the combined impact of anxiety and depression (comorbidity) on parenting behaviors during the perinatal period is limited. This comorbidity is associated with a higher risk of adverse outcomes for both mothers and children, including poor neonatal outcomes and cognitive, emotional, or behavioral outcomes during childhood [30, 31]. Additionally, it may further, exacerbate emotional dysregulation and parenting difficulties, [32–34], including inconsistent parenting behaviors due to the cumulative effects of heightened worry, decreased energy, and emotional withdrawal [35, 36]. Moreover, comorbid conditions further, compromise parent's ability to respond sensitively to their child's cues, interfering in the caregiving environment and amplifying the risk of adverse developmental outcomes [37, 38].

The dimensions of guided learning, protection, and control offer a valuable framework for examining the relationship between perinatal mental health and parenting behaviors. A major challenge is to synthesize the existing literature given the variability in methodologies used to assess anxiety, depression, and parenting behaviors [39]. Studies differ in terms of measures (self-report versus clinical interviews) for mental health assessment, as well as in terms of methods (observational versus self-report) for evaluating parenting behaviors [40]. Additionally, there is considerable heterogeneity in how parenting behaviors and dimensions are conceptualized, with many studies lacking a consistent organizational structure. This variability compromises direct comparisons and limits the ability to draw conclusions about the effects of anxiety and depression, both individually and in combination, on parenting behaviors [41].

To address these methodological and conceptual challenges, we categorize parenting behaviors into these three dimensions. Regardless of the original framework used, we aim to generate a more integrated and comparable understanding of the association between perinatal anxiety, depression, and their comorbidity and parenting behavior. In summary, this systematic review aims to assess the (1) associations between perinatal anxiety and depressive symptoms and parenting behaviors across the dimensions of protection, control, and guided learning, and (2) associations between anxiety-depression comorbidity and parenting behaviors within these dimensions.

While both mothers and fathers can experience anxiety and depressive symptoms during the transition to parenthood, this review focuses solely on mothers, due to the established evidence base linking maternal mental health to early parenting behaviors and child outcomes [30, 42]. Although family structures have undergone significant changes globally, mothers are often regarded as the primary caregivers during the early postpartum years across many cultural contexts [43–45]. As a result, maternal parenting behaviors are more frequently studied and have been strongly linked to infant developmental outcomes. Second, women are more likely to experience perinatal anxiety and depression compared to men [46, 47], and can be more prone to inconsistent parenting behaviors that compromise the caregiving environment [25, 27].

## 2. Method

### 2.1. Protocol Registration

The protocol for this review was registered in April 2023 in the PROSPERO international prospective register of systematic reviews (CRD42023337333). Deviations from the original protocol included not conducting the planned meta-analysis, as an insufficient number of studies reported comorbidity between anxiety and depressive symptoms, and data requests from study authors did not allow for a methodologically valid analysis. The procedures followed the Preferred Reporting Items for Systematic Reviews and Meta-Analyses (PRISMA) guidelines [48].

### 2.2. Literature Search

The literature search was conducted in January 2024, with no date restrictions. The search was performed across four electronic databases: Web of Science, SCOPUS, PUBMED, and EBSCO. The search terms used were: (“anxi*⁣*^*∗*^” AND “depress*⁣*^*∗*^”) AND (“parent*⁣*^*∗*^” OR “mother–infant interaction” OR “parent–infant interaction” OR “mother–child interaction” OR “parent–child interaction” OR “mother–toddler interaction” OR “parent–toddler interaction”) AND (pregnancy OR antenatal OR postnatal OR postpartum OR prenatal OR gestation). The full search query for each database can be found in Supporting Information 1: Table S1. No restrictions were applied.

### 2.3. Eligibility Criteria

Eligibility criteria were established using the PECOS framework [49]. The population of interest (*P*) included pregnant women and mothers within the first 3 years postpartum. The exposure of interest (*E*) was perinatal anxiety and/or depression, and studies were included if they presented data on the association between anxiety and depressive symptoms and/comorbid anxiety-depressive symptoms and parenting behaviors. Parenting behavior had to be assessed using standardized observational or self-report measures. The outcome of interest (*O*) was parenting behavior during the first 3 years postpartum (e.g., sensitivity, responsiveness, control, intrusiveness, warmth, feeding, etc.). No specific comparison (*C*) group was required, as the focus was on examining the abovementioned associations in a correlational fashion. Eligible studies were required to use quantitative designs (*S*), including cross-sectional, case-control, longitudinal studies, or randomized controlled trials, and to be published in peer reviewed journals. Studies were excluded if they were dissertations, theses, systematic reviews, meta-analysis, literature reviews, protocols, research projects, books, book chapters, or conference proceedings.

### 2.4. Information Extracted for the Systematic Review

Two researchers (AM and RP) screened independently databases by titles and abstracts for potentially eligible studies. Disagreements between researchers were resolved by consensus or arbitration involving a third author when necessary. Full texts of studies were retrieved and assessed for inclusion/exclusion criteria. Researchers extracted and coded information data from the included studies. Discrepancies were resolved through consensus meetings, and a third rater reviewed 10% of the studies to minimize bias. Data extracted included maternal characteristics, such as age, education, marital status, and parity. Infant age categories were grouped into 0–6 months, 6–12 months, and 12–36 months to provide a developmental framework for interpreting the results. Study characteristics, including country, sample size, sample type, study design, number of assessments, and the measures used to assess perinatal anxiety, depression, and parenting behavior, were also extracted.

The specific parenting behaviors/constructs (e.g., sensitivity and hostility) were extracted and coded according to the definitions provided by the authors. In cases where the authors did not provide definitions, we referred to the original validation studies of the measures used and applied the definitions provided for the parenting behavior/construct. The behaviors were classified into the three parenting dimensions: protection, control, and guided learning [18]. Information (e.g., specific measures) not reported was coded as “nonavailable” (N/A).

### 2.5. Quality and Risk of Bias Assessment

The quality of the studies was assessed independently by eight coders randomly divided into pairs, using the 12-question (14 items) critical appraisal skills programme (CASP) [50] checklist for cohort studies. The CASP checklist evaluates the validity, results, and local applicability of the study. The scoring system is as follows: “no” scored as 0, “partially agree” as 0.5, and “yes” as 1. Studies were categorized as high quality if scored between 11 and 14 points, moderate quality for scores between 8 and 10 points, and low quality for scores below 8 points [51]. Disagreements were resolved through consensus meetings with the first author.

## 3. Results

### 3.1. Study Selection and Included Studies

**Figure 1:**
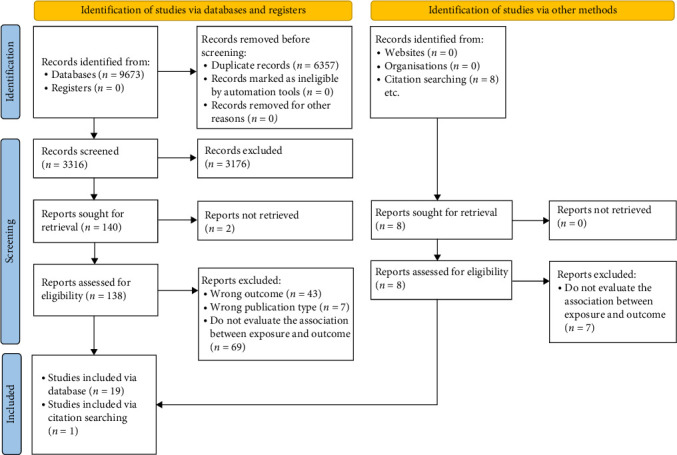
PRISMA flow diagram of study selection process.

A total of 9673 potentially relevant documents from databases were identified and imported into Rayyan [52] for screening. After removing duplicates (*n* = 6357), 3316 records were screened by title and abstract, resulting in 138 full-text articles for review. In addition, eight potentially relevant documents were identified from websites and assessed for eligibility. In totality, 20 studies met the inclusion criteria for the systematic review. The study selection process, including reasons for exclusion at each stage, is depicted in the PRISMA flow diagram (see Figure 1).

### 3.2. Sample and Study Characteristics

The 20 studies included 2426 participants, with sample sizes ranging from 24 [53] to 647 participants [54]. The mean age of the mothers across studies was 31.93 years (*SD* = 4.93). Infants' ages ranged from 0 to 36 months, with 65% (*n* = 13) under 6 months of age. Data on marital status was reported in 14 studies (70%), with 12 studies indicating that participants were predominantly married or cohabiting. The percentage of married participants ranged from 64% to 100% across studies; parity was reported in 12 studies (60%) of these, one study (8.3%) included only primiparous women. The remaining 11 studies included both primiparous and multiparous women, with the proportion of primiparous women ranging from 35% to 88.3%, and the proportion of multiparous women ranging from 11.8% to 78%. No study provided information on the number of children among multiparous women. Educational level was reported in 90% of studies (*n* = 18), with about 77.8% of participants having attained higher education (see Table 1).

The included studies were conducted in European countries (*n* = 8), United States (*n* = 7), and Australia (*n* = 4). Most of them used community-based samples (75%; *n* = 15), while 25% (*n* = 5) clinical samples. More than half of studies had a longitudinal design (*n* = 11; 55%), with 63.6% of these having two assessment waves (*n* = 7). Of the longitudinal studies, 72.7% (*n* = 8) had a baseline assessment during pregnancy, while 27.3% (*n* = 3) had baseline assessment during the postpartum period (see Table 2). Nine studies (45%) had a cross-sectional design.

### 3.3. Risk of Bias

Eleven studies (55%) were assessed as having a low overall risk of bias, while nine studies (45%) presented a moderate risk, and no study was categorized as high risk. Regarding specific domains of bias, 65% of studies (*n* = 13) reported adequate sample recruitment, and 25% (*n* = 5) had sufficiently complete follow-up data. Outcome measurement to minimize bias was adequate in 95% of studies (*n* = 19), while 50% (*n* = 10) demonstrated findings generalizable to the local population (see Supporting Information 2: Table S2).

### 3.4. Assessment of Perinatal Anxiety and Depressive Symptoms

Anxiety symptoms were assessed using eight distinct self-report measures, with the State–Trait Anxiety Inventory (STAI-T/S; [72]) being the most frequently utilized (40% of the studies; *n* = 8). This measure captures both chronic (trait) and situational (state) anxiety, with some studies utilizing only the state subscale (STAI-S; 10%, *n* = 2) and others administering exclusively on the trait subscale (STAI-T; 10%, *n* = 2). The Generalized Anxiety Disorder Questionnaire (GAD-Q; [73]) was used in 15% of studies (*n* = 3), while the Symptom Checklist (SCL; [74]) was employed in 10% of studies (*n* = 2).

For depressive symptoms, 10 different self-report measures were used. The Edinburgh Postnatal Depression Scale (EPDS; [75]) was the most widely used, appearing in 55% of the studies (*n* = 11). The Center for Epidemiological Studies-Depression Scale (CES-D; [76]) was used in 20% of the studies (*n* = 4). The EPDS is specifically designed to assess postpartum depressive symptoms, whereas the CES-D measures general depressive symptoms (see Table 2). There was considerable variation in the period of assessment for anxiety and depressive symptoms. Eight studies (40%) assessed these symptoms during pregnancy, while 12 studies (60%) focused exclusively on the postpartum period.

### 3.5. Assessment of Parenting Behaviors

Eleven different observational measures were used to assess parenting behaviors, focusing mainly on mother-infant interactions during free play (*n* = 18; 90%). All studies used observational measures to evaluate parenting behavior. The CARE-Index [77] was the most frequently utilized, being used in 25% of studies (*n* = 5), followed by the Emotional Availability Scales (EA-S; [78]), used in 20% of studies (*n* = 4). One study used their own coding systems to evaluate the free play interaction and code parenting behavior [70]. Across studies, 17 distinct parenting behaviors were assessed and categorized into the three primary dimensions of protection, control, and guided learning, in accordance with the Davies et al. [18] model. Sensitivity was the most frequently assessed behavior in the protection dimension, controlling the most frequently assessed behavior in the control dimension, and intrusiveness the most frequently assessed in the guided learning dimension (see Table 2).

### 3.6. Overview of the Associations Between Perinatal Anxiety or Depressive Symptoms and Parenting Dimensions

Of the 20 studies included in this review, 18 (90%) found association between either anxiety or depressive symptoms and at least one of the three parenting dimensions during the perinatal period. Specifically, 15 studies (75%) reported association between anxiety symptoms and at least one parenting dimension, while 15 studies (75%) reported association between depressive symptoms and at least one parenting dimension. When analyzing the associations between anxiety or depressive symptoms and specific parenting dimensions, 15 studies (75%) identified an association between either anxiety or depressive symptoms and the protection dimension. Four studies (20%) reported an association between either anxiety or depressive symptoms and the guided learning dimension, while four studies (20%) found an association between either anxiety or depressive symptoms and the control dimension.

### 3.7. Associations Between Perinatal Anxiety Symptoms and Parenting Dimensions

#### 3.7.1. Parental Protection

Nineteen studies (95%) examined the relationship between anxiety symptoms and parenting behaviors classified under the protection dimension. Of these, eight studies (42.1%) reported no significant association between anxiety symptoms and parenting behaviors within this dimension (e.g., [61]). However, among the remaining 11 studies, six (31.6%) identified associations between anxiety symptoms and all parenting behaviors classified under the protection dimension assessed in the study (e.g., [62]) while five studies (26.3%) reported mixed findings, with associations observed for some behaviors within this dimension but not others (e.g., [70]).

Regarding the assessment period of anxiety symptoms and parenting behaviors in the 11 studies that identified at least one association between anxiety symptoms and one parenting behavior within the protection dimension, two studies reported associations with prenatal anxiety symptoms [42, 53], one study reported associations with pre and postnatal anxiety symptoms [62], while eight studies identified associations exclusively during the postpartum period (e.g., [63]).

The most frequently studied parenting behaviors within the protection dimension were maternal sensitivity and responsiveness. Maternal sensitivity was examined in 15 studies (78.9%), of which seven (46.7%) reported significant associations between higher anxiety symptoms and lower sensitivity (e.g., [59]). Maternal responsiveness was investigated in nine studies (47.3%), with three (33.3%) identifying significant associations between higher anxiety symptoms and lower responsiveness [53, 70].

#### 3.7.2. Parental Control

Eight studies (40%) examined the relationship between anxiety symptoms and parenting behaviors classified under the control dimension. Of these, five studies (62.5%) reported no significant associations within this dimension (e.g., [68, 70]). Among the remaining three studies (37.5%), all identified associations between anxiety symptoms and all parenting behaviors classified under the control dimension assessed in the study [42, 56, 64]. Regarding the assessment period, one study reported associations between prenatal anxiety symptoms and parental control behaviors [42], while two studies identified associations during the postnatal period [56, 64].

The most frequently studied parenting behavior within the control dimension was controlling. Controlling was examined in five studies (62.5%), of which three (60%) identified significant associations between anxiety symptoms and higher maternal controlling [42, 56, 64].

#### 3.7.3. Parental Guided Learning

Eight studies (40%) investigated the relationship between anxiety symptoms and parenting behaviors categorized under the guided learning dimension. Of these, five studies (62.5%) reported no significant association between anxiety symptoms and parenting behaviors within this dimension (e.g., [67]). Among the remaining three studies (37.5%), two (66.7%) found associations with all parenting behaviors within the guided learning dimension assessed [54, 60], while one (33.3%) reported mixed findings, with associations identified for some behaviors but not others [58].

In terms of the assessment period, only one observed associations between prenatal anxiety symptoms and guided learning behaviors [60], while two studies identified associations exclusively during the postpartum period [54, 58].

The most frequently studied parenting behavior within the guided learning dimension was intrusiveness. Six studies (75%) investigated maternal intrusiveness, with three (50%) reporting significant associations between elevated anxiety symptoms and increased maternal intrusiveness [54, 58, 60].

### 3.8. Associations Between Perinatal Depressive Symptoms and Parenting Dimensions

#### 3.8.1. Parental Protection

Eighteen studies (90%) examined the relationship between depressive symptoms and parenting behaviors classified under the protection dimension. Of these, six (33.3%) reported no significant association within this dimension (e.g., [42]). Among the remaining 12 studies, seven (38.9%) identified associations across all parenting behaviors assessed (e.g., [66]), while five (27.8%) reported mixed findings, with associations observed for some behaviors but not others (e.g., [70]).

When considering the assessment period, two studies reported associations with prenatal depressive symptoms (e.g., [57]), one study reported associations with pre and postnatal depressive symptoms [62], while eight identified associations exclusively during the postpartum period (e.g., [69, 70]).

Similar to findings on anxiety symptoms, sensitivity and responsiveness were the most frequently studied parenting behaviors within the protection dimension in the associations with depressive symptoms. Maternal sensitivity was examined in 15 studies (83.3%), with six (40%) reporting significant negative associations with depressive symptoms (e.g., [64]). Responsiveness was investigated in nine studies (50%), of which three (33.3%) identified associations between higher depressive symptoms and lower responsiveness (e.g., [53, 70]).

#### 3.8.2. Parental Control

Eight studies (40%) investigated the associations between depressive symptoms and parenting behaviors classified under the control dimension. Of these, three studies (37.6%) identified associations between depressive symptoms (all assessed during the postnatal period) and all parenting behaviors analyzed [56, 60, 68], while five studies (62.5%) reported no significant associations (e.g., [42, 62]). Controlling behaviors were the most frequently examined parenting behaviors within this dimension, assessed in four studies, with two studies identifying significant associations between higher depressive symptoms and greater maternal controlling (e.g., [56]).

#### 3.8.3. Parental Guided Learning

Seven studies (35%) examined the association between depressive symptoms and parenting behaviors classified under the guided learning dimension. Of these, four studies (57.1%) reported no significant association between depressive symptoms and parenting behaviors within this dimension (e.g., [71]). Among the remaining three studies (42.9%), one study (14.1%) identified associations between depressive symptoms and all parenting behaviors assessed in the study [60], while two studies (28.8%) reported mixed findings, with associations observed for some behaviors within this dimension but not others [54, 67].

Regarding the assessment period of depressive symptoms and parenting behaviors in the five studies that identified at least one association, one study reported an association with prenatal depressive symptoms and guided learning behaviors [60], while other two studies identified associations exclusively during the postpartum period [54, 67]. Intrusiveness was the most frequently studied parenting behavior within the guided learning dimension, assessed in four studies, of which two studies identified significant associations between depressive symptoms and maternal intrusiveness [54, 60].

### 3.9. Comorbidity of Perinatal Anxiety and Depressive Symptoms and Parenting Behaviors

Two studies (10%) examined the association between comorbidity of anxiety and depressive symptoms and parenting behaviors [55, 58]. Aran et al. [55] also found that postnatal comorbid anxiety and depressive symptoms was associated with the protection dimension, in specific to emotionally unavailable. Dib et al. [58] also reported that comorbidity assessed during the postnatal period was associated with parenting behaviors under the protection dimension (i.e., lower maternal sensitivity), and behaviors under guided learning dimension, namely, lower cognitive stimulation and greater disengagement, and intrusiveness.

## 4. Discussion

In this systematic review, we aimed to investigate the differential associations between perinatal anxiety and depressive symptoms and parental protection, control, and guided learning [17, 18]. Additionally, we aimed to address the associations between anxiety-depression comorbidity and parenting behaviors within these dimensions.

### 4.1. Parental Protection

Maternal sensitivity and responsiveness behaviors were more frequently explored in the presence of perinatal anxiety and depressive symptoms, with a higher percentage of studies finding an association between perinatal anxiety and depressive symptoms and difficulties in maternal sensitivity. Mothers experiencing anxiety symptoms may exhibit hypervigilance and intrusive behaviors that compromise their ability to respond sensitively to their infant's emotional cues [79]. These behaviors may be driven by an exaggerated perception of potential threats to the child's safety, leading the mother to become overly involved in the child's activities [64].

Similarly, mothers experiencing depressive symptoms, which involve the presence of cognitive, affective, and physical symptoms [80, 81], may display emotional disengagement, which can hinder their ability to provide the necessary emotional support for fostering secure attachment [62]. This emotional unavailability is rooted in cognitive distortions, such as negative self-appraisals, rumination, and learned helplessness [82, 83]. These cognitive patterns, coupled with anhedonia, may lead to a reduction in positive interactions, and responsiveness to the child's emotional needs, as mothers may find it difficult to recognize child cues and experience joy or satisfaction in caregiving [84].

Given the critical role of protection-related parenting behaviors during the first 3 years of life, robust and consistent associations with maternal anxiety and depressive symptoms would be expected. However a considerable proportion of studies reported null findings for both depressive and anxiety symptoms, highlighting variability that warrants closer examination.

One possible explanation for these null findings is the variability in measurement approaches across studies. Many studies in our systematic review used general measures of protection-related parenting behaviors, such as free play tasks, which are not specifically designed to assess protective behaviors in distress-related contexts (e.g., [70]). Protective caregiving, as conceptualized within the protection dimension, is activated in response to child distress and is essential for fostering safety and emotional security [16]. The use of broad or nonspecific measures may have contributed to variability in findings, potentially affecting the ability to detect associations between maternal mental health symptoms and protection-related behaviors. While some studies employed observational tools, such as the EA-S [78] or Coding Interactive Behavior [85], these tools are flexible in their application and can be used in both distress-specific and emotionally neutral caregiving contexts. When applied in neutral contexts, such as free play, they might be less likely to capture core parenting behaviors under this dimension. Consequently, it remains unclear whether the variability in findings reflects true differences in parenting behaviors or methodological inconsistencies across studies. Future research should prioritize the systematic use of domain-specific tools explicitly designed to assess protection-related parenting behaviors in distress contexts.

Another factor influencing the variability in findings may be differences in symptom severity across study populations. The meta-analysis by Bernard et al. [86] reported stronger associations between maternal depression and sensitivity in clinical samples with higher symptom severity compared to community-based samples with subclinical symptoms, suggesting that higher symptom severity amplifies the effects of mental health symptoms on caregiving behaviors. However, in our systematic review, null findings were observed across both clinical and community-based samples, indicating that symptom severity alone does not fully account for the variability in results. Other factors, such as heterogeneity in measurement approaches and caregiving contexts, could likely contribute to these inconsistencies. Additionally, Bernard et al.'s meta-analysis exclusively focused on sensitivity within the first year of life, whereas our review included studies of children up to 3 years of age. This broader developmental focus may contribute to the null findings, as the salience of protection-related parenting behaviors diminishes over time. Protection is most relevant during infancy when children rely heavily on caregivers for emotional regulation and coping with threats [16–18]. As children develop greater emotional regulation capabilities and distress signals become less pronounced, the prominence of protection-related parenting behaviors may decline, introducing variability in findings across studies that span a wider developmental age range.

The variability in findings may also stem from the absence of contextual moderators in the analytic models of many included studies. Without accounting for these factors, studies may underestimate the buffering effects of social or relational moderators, leading to null findings even when maternal anxiety or depressive symptoms are present. Potential moderators, such as maternal demographics, marital quality, infant regulatory capacity, and coparenting relationship are theoretically grounded and warrant further, investigation to clarify the nature of these null findings [87–89].

The small but significant negative association between maternal depression and sensitivity reported in Bernard et al. [86] contextualizes the moderate prevalence of null findings observed in our systematic review. Bernard et al. [86] attributed this small effect to methodological inconsistencies, such as reliance on community-based samples and single-task designs. Similar issues in our work, including the variability in measurement tools, may account for variability in results observed in this dimension. These findings underscore the need for future research to adopt domain-specific measures tied to distress-related contexts and to systematically investigate key moderators to better understand the relationship between perinatal mental health symptoms and protection-related parenting behaviors.

### 4.2. Parental Control

Our review found that most studies did not report significant associations between perinatal anxiety or depressive symptoms and parental control behavior. Control-related behaviors, including boundary-setting, structuring, and discipline, appear to be less central than protection-focused behaviors during the first 3 years of life but gain increasing relevance as children's autonomy and self-regulation develop. Early parenting is primarily oriented toward ensuring safety and fostering emotional security, which aligns more closely with the protection dimension [16, 18]. As children begin to acquire motor and cognitive skills, the developmental relevance of parental control behaviors increases, becoming more prominent as autonomy and self-regulation emerge in later stages [90, 91]. In infancy and toddlerhood, however, children's limited capacities reduce the need for explicit regulatory behaviors, such as boundary enforcement or conflict resolution. During this period, parental control strategies are typically characterized by subtle scaffolding and guidance rather than overt disciplinary actions, further, limiting the variability observed in control-related behaviors.

Another contributing factor to these findings may be the variability in how parental control behaviors were defined and measured across studies. Constructs, such as structuring and harsh parenting were assessed using diverse tools applied to varying caregiving contexts [55, 70]. This methodological heterogeneity might complicate comparisons across studies and may reduce the sensitivity needed to detect associations with perinatal anxiety and depressive symptoms. For instance, while some studies employed broad observational tools to capture more general control-related behaviors and caregiving practices, others focused on specific regulatory strategies [68]. Such inconsistency in measurement approaches may have diluted the capacity to detect associations, contributing to the predominance of null findings in this dimension. Future research should prioritize the development of standardized and context-specific measures, grounded in evolutionary-developmental frameworks, to ensure that null findings accurately reflect the absence of associations between anxiety and depressive symptoms and parental control dimension, rather than being an artifact of methodological limitations.

### 4.3. Parental Guided Learning

Similarly to the control dimension, while null findings were more frequent, a substantial minority of studies identified associations between perinatal anxiety or depressive symptoms and guided learning behaviors (e.g., [58, 59, 67, 69]). A few studies reported significant associations, particularly for anxiety-related intrusive behaviors [54, 60], while most findings remained null across the behaviors examined. For behaviors, such as engagement, involvement, affect, stimulation, emotional regulation, remoteness, and task-oriented behaviors, most studies did not detect associations with anxiety symptoms (e.g., [58, 67]). However, some studies found effects on specific behaviors, particularly intrusiveness [54, 60]. Although, theoretical models suggest that mothers with anxiety symptoms may display hypervigilance and intrusive behaviors, potentially impairing their ability to guide their infant's emotional cues [79], empirical findings reported in this review were inconsistent. Some studies supported this hypothesis (e.g., [54, 59, 60]), while others failed to detect significant associations (e.g., [58, 67]). Similarly, for depressive symptoms, evidence was mixed. While most studies did not detect significant associations, a subset of studies reported effects, particularly on intrusiveness, with disengagement observed in fewer cases [58, 67, 69]. The variability in findings suggests that while guided learning behaviors may be less central during early infancy, certain aspects – particularly those related to parental control and responsiveness – may still be sensitive to maternal depressive and anxiety symptoms. The predominance of null findings may, therefore, reflect the limited developmental salience of guided learning behaviors during the first 3 years of life rather than a complete absence of influence.

Guided learning, which involves parental support for the development of children's cognitive, physical, social, or emotional skills, typically occurs through structured interactions, such as play sessions or educational tasks [16]. Notably, almost all studies evaluating guided learning in this review focused on interactions with infants aged 6 months or younger. At this stage, children's cognitive and exploratory capacities are still in their initial phases of development, reducing the necessity for complex scaffolding behaviors and structured support. Consequently, structured guided learning interactions may be less frequent, particularly in free play settings, where opportunities for observing explicit parental scaffolding are limited.

While structured guided learning interactions may be less frequent in early infancy, some studies found that specific guided learning behaviors, such as intrusiveness and cognitive stimulation, were still present and associated with maternal symptoms (e.g., [58, 59, 67, 69]). Parents may also adapt their behaviors to align with their child's developmental limitations, further, reducing the variability and intensity of guided learning behaviors during infancy and toddlerhood. As children acquire cognitive and linguistic skills, guided learning behaviors become more prominent, enabling them to engage in collaborative problem-solving and more structured tasks [16–18]. This shift suggests that guided learning assumes greater importance during later developmental stages, such as early childhood, when parenting demands increasingly involve supporting learning and cognitive development (e.g., [92]). Consequently, while null findings were more prevalent, a meaningful proportion of studies identified associations between perinatal mental health symptoms and guided learning behaviors in early infancy, particularly in relation to intrusiveness and cognitive stimulation. This suggests that while guided learning may be less central to parenting in this period, specific behaviors related to parental responsiveness and control may be influenced by maternal anxiety and depressive symptoms.

### 4.4. Perinatal Anxiety and Depressive Comorbidity

Our findings highlight the scarcity of research examining the association between comorbid perinatal anxiety and depressive symptoms and parenting behaviors, with only two studies investigating this relationship [55, 58]. Both studies identified significant associations, but the available evidence remains limited and preliminary. Aran et al. [55] found that postnatal comorbid anxiety and depressive symptoms were associated with lower maternal emotional availability, suggesting that co-occurring symptoms may exacerbate difficulties in maintaining responsive and emotionally engaged interactions with the infant. Dib et al. [58] further, demonstrated that comorbidity was linked to both reduced protection and guided learning behaviors, specifically lower maternal sensitivity, decreased cognitive stimulation, and increased disengagement and intrusiveness.

While these findings align with broader research in psychopathology suggesting that co-occurring mental health problems may intensify emotional dysregulation and cognitive difficulties, the limited number of studies prevents drawing robust conclusions. However, the findings suggest that co-occurring symptoms may be linked to reduced maternal sensitivity, lower cognitive stimulation, and increased disengagement and intrusiveness. Given that only two studies examined this association, further, research is needed to determine whether comorbidity exerts distinct effects beyond the independent influences of anxiety and depression.

### 4.5. Methodological Strengths and Limitations

This systematic review has several methodological strengths. By incorporating studies that assess both anxiety and depressive symptoms allowed us to better explore the association between these symptoms and parenting behaviors. If we had only included studies that focused on one condition, we would have missed the opportunity to explore these differential associations, which is a methodological strength of this review. This approach provided a more robust analysis of the differential association of these symptoms, allowing us to explore not only their independent association but also their potential combined impact. Most included studies had a low or moderate risk of bias, with none categorized as high risk. While outcome measurement strategies were generally adequate, limitations in sample recruitment and incomplete follow-up data may have influenced the reliability of findings.

A significant strength of this review is the inclusion of 13 longitudinal studies spanning the prenatal and postnatal periods, while some focused exclusively on the postnatal period. Longitudinal allow for a temporal examination of maternal mental health symptoms and their associations with parenting behaviors over time. Additionally, the exclusive use of observational measures of parenting behaviors provides objective and ecologically valid assessments, reducing biases associated with self-reported data. Importantly, this review employed an evolutionary-developmental framework to organize parenting behaviors into protection, control, and guided learning dimensions [16–18]. This theoretical lens provided a consistent structure for synthesizing findings across diverse studies and revealed stronger associations between maternal mental health symptoms and protection behaviors compared to control or guided learning dimensions.

However, limitations should also be noted. The variability in self-report measures for assessing mental health symptoms, as well as differences in the timing of these assessments, which may have introduced inconsistencies in the findings. Specifically, differences in the sensitivity and specificity of various mental health assessment tools can lead to varying levels of symptom detection [93, 94]. This heterogeneity in measurement tools and study designs may reflect differences in how anxiety and depressive symptoms are conceptualized and assessed across studies, limiting the comparability of results and complicating the synthesis of findings. These issues underscore the importance of adopting standardized, validated measures to enhance the interpretability and generalizability of future research. Additionally, the included studies exclusively used self-report measures without incorporating other forms of assessment like clinical interviews, which may have limited the sensitivity and precision of the assessment of anxiety and depressive symptoms. Another limitation of this systematic review is its exclusive focus on mothers, despite growing evidence underscoring the significant role of fathers' anxiety and depressive symptoms and parenting behaviors [95]. Future systematic reviews should include both maternal and paternal anxiety and depressive symptoms to better understand how their interactions are associated with parenting behaviors in both mothers and fathers.

Finally, although methodological differences in measurement tools, study designs, and sample characteristics (e.g., primiparous versus multiparous participants) may have contributed to variability in findings, they do not fully explain the observed heterogeneity. These limitations highlight the need for greater methodological rigor and consistency in future research to enhance the reliability of findings and advance our understanding of the associations between perinatal mental health and parenting behaviors.

### 4.6. Clinical Implications

Our findings emphasize the need for targeted interventions that address the differential impact of perinatal anxiety and depressive symptoms on parenting behaviors during the first 3 years of life. Protection-related behaviors, particularly maternal sensitivity and responsiveness, show the strongest associations with these symptoms, underscoring the importance of fostering emotional availability in caregiving. Given these findings, implementing specific actions must be considered.

Screening for mental health symptoms, combined with assessments of protection-related parenting behaviors during prenatal and postnatal care, could facilitate early identification of at-risk mothers and support the timely implementation of interventions focused on enhancing maternal sensitivity and responsiveness.

The variability in findings regarding the timing of symptom assessment highlights the need for clinical strategies that span both prenatal and postpartum periods. Prenatal symptoms may impact long-term caregiving behaviors, while the postpartum period presents immediate caregiving demands, which may amplify the influence of maternal mental health symptoms. Clinical care should integrate screenings throughout the perinatal period, ensuring early identification of risks during pregnancy and delivering targeted support tailored to the demands of the postpartum period.

### 4.7. Future Directions

Although associations were observed in the protection dimension of parenting, the predominance of null findings, especially in the control and guided learning dimensions, indicates the need for more focused research. One important avenue is the examination of comorbid anxiety and depressive symptoms during the perinatal period. Most studies to date have treated these symptoms as separate constructs, overlooking how their overlap might influence parenting behaviors. Using person-centered methods, such as latent class analysis, could identify distinct symptom profiles that better capture the variability and interaction between these symptoms. Such an approach would provide a more precise understanding of how co-occurring symptoms impact specific parenting dimensions and inform the development of tailored interventions. Another critical avenue for future research is to gather more robust longitudinal data to elucidate the temporal patterns of how parental anxiety and depressive symptoms influence parenting behaviors across the perinatal period. Specifically, studies should investigate whether symptoms experienced during the prenatal period have enduring effects on postpartum parenting behaviors or whether postpartum symptoms exert a more immediate and direct influence. Understanding these temporal patterns is essential to identifying optimal periods for targeted intervention.

Contextual factors, such as parity, should be systematically examined as a potential moderator. Parents with caregiving experience may draw on established strategies that buffer the effects of mental health symptoms, whereas first-time parents may encounter greater challenges in adapting to parenting demands in the presence of anxiety or depression [96].

Finally, greater standardization in how mental health symptoms and parenting dimensions are conceptualized and measured is essential. Current variability in measurement tools complicates comparisons across studies and limits the ability to draw clear conclusions. Future research should adopt validated, theory-driven tools that align with established frameworks, particularly those grounded in evolutionary-developmental principles. These tools should be designed to capture the nuanced and context-specific nature of parenting behaviors, especially in domains like control and guided learning, which may not be as prominent during the first 3 years of life. Standardizing these approaches would allow for more robust evaluations of how perinatal mental health symptoms influence caregiving behaviors.

### 4.8. Conclusions

Associations between perinatal anxiety and depressive symptoms and parenting behaviors were more frequently observed in the protection dimension, particularly in relation to maternal sensitivity and responsiveness. In contrast, null findings were more prevalent in the control and guided learning dimensions. While this may reflect their lower developmental salience during the first 3 years of life, further, research is needed to confirm whether methodological factors also contribute to these patterns. These findings indicate that protection-related behaviors may be particularly relevant for interventions targeting perinatal mental health, while future research should examine how parenting challenges in control and guided learning evolve as children develop autonomy and cognitive skills.

Despite these insights, significant gaps in the literature remain. The role of comorbid anxiety and depressive symptoms, variability in the measurement and conceptualization of parenting dimensions, and the long-term influence of prenatal versus postpartum symptoms are critical areas requiring further investigation. Addressing these gaps through longitudinal designs and standardized measures will advance our understanding of how perinatal mental health symptoms impact parenting and support the development of tailored, evidence-based interventions.

## Figures and Tables

**Table 1:** Detailed demographics of included studies (*N* = 20).

Authors Country	Setting	Mothers sample (*N*) and mothers' age at baseline (*M*, *SD*)	Months	Education	Married/partner (%)	Parity (multiparous versus primiparous)
Aran et al. [55] Australia	Clinical	*N* = 115 Group 1: *M* = 33.66, *SD* = 5.07 Group 2: *M* = 34.01, *SD* = 4.76	0–6; 6–12	College degree: 57%	93%	54% Multiparous; 46% primiparous

Crugnola et al. [56] Italy	Clinical	*N* = 73 (*M* = 33.55, *SD* = not reported)	0–6	Professional certificate or diploma: 55.4%	95.5%	22% Multiparous; 78% primiparous

Della Vedova et al. [57] Italy	Community	*N* = 43 (*M* = 31.81, *SD* = 3.33)	0–6	High school/college degree: 81.4%	76.7%	All primiparous

Dib et al. [58] Brazil	Clinical	*N* = 40 (≥25 years = 72.5%)	6–12; 12–36	Majority with high school diploma (% not reported)	87.5%	65% Multiparous; 35% primiparous

Ferber and Feldman [59] Israel	Community	*N* = 81 (*M* = 33.42, *SD* = 5.04)	0–6	College degree: 49.38%	100%	64.2% Multiparous; 35.8% primiparous

Hakanen et al. [60] Finland	Community	*N* = 190 (*M* = 31.15, *SD* = 4.07)	0–6	Middle education level: 38.3%	N/A	43.1% Multiparous; 56.9% primiparous

Hart et al. [61] USA	Community	*N* = 50 Group 1: *M* = 27.61, *SD* = 4.57 Group 2: *M* = 27.89, *SD* = 4.26	6–12	11–15 years	76%	64% Multiparous; 36% primiparous

Holmberg et al. [62] Finland	Community	*N* = 103 (*M* = 31.04, *SD* = 4.51)	6–12; 12–36	College degree: 41.7%	N/A	48.6% Multiparous; 51.4% Primiparous

Ierardi et al. [63] Italy	Community	*N* = 70 (*M* = 34.59, *SD* = 5.21)	0–6	*M* = 15.22 years	100%	N/A

Ierardi et al. [64] Italy	Community	*N* = 81 (*M* = 33.42, *SD* = 5.04)	0–6	High-school: 52.1%	96%	N/A

Kaplan et al. [65] USA	Community	*N* = 47 (*M* = 28, *SD* = not reported)	12–36	N (A)	N/A	N/A

Keren et al. [66] Israel	Clinical	*N* = 47 (*M* = 28.60, *SD* = 6.09)	0–6	*M* = 13.66 years	N/A	N/A

Neri et al. [67] Italy	Community	*N* = 197 Group 1: *M* = 33.54, *SD* = 5.46 Group 2: *M* = 33.82, *SD* = 5.91 Group 3: *M* = 32.89, *SD* = 5.02	0–6	Group 1:11.90 years Group 2:12.78 years Group 3:14.67 years	Group 1:96.9%; Group 2:97.8%; Group 3:92.3%	Multiparous; primiparous: Group 1:25%; 75.0% Group 2:33.3%; 66.7% Group 3:11.7%; 88.3%

Ojo et al. [68] USA	Community	*N* = 133 (*M* = 22.2, *SD* = 5.7)	6–12	Middle/high school: 29%	63%	20% Multiparous; 80% primiparous

Parfitt et al. [42] UK	Community	*N* = 44 (*M* = 33.12, *SD* = 4.79)	0–6	College degree: 97%	64%	N/A

Sandre et al. [69] Canada	Community	*N* = 58 (*M* = 33.10, *SD* = 4.54)	0–6	N/A	N/A	N/A

Stein et al. [70] USA	Community	*N =* 253 (*M =* 32.7, *SD =* 4.84)	0–6; 6–12	College degree: 60.9%	N/A	39.5% Multiparous; 60.5% Primiparous

Tluczek et al. [71] USA	Clinical	*N* = 130 (*M* = 29.72 *SD* = not reported)	6–12	College degree: 40.0%	83.1%	N/A

Warnock et al. [53] Canada	Community	*N* = 24 (*M* = 34, *SD* = 5.6)	0–6	*M* = 17.46	63%	37% Multiparous; 63% Primiparous

Weiss et al. [54] USA	Community	*N* = 647 (*M* = 31.3, *SD* = 5.9)	6–12	College degree: 63%	87.5%	N/A

*Note:* Infant's ages were grouped into the following categories: 0–6 months, 6–12 months, and 12–36 months.

Abbreviation: N/A, nonavailable.

**Table 2:** Detailed measures and main of included studies (*N* = 20).

Authors Country	Setting	Study design	(N° of wave assessments) Timing of assessments – prenatal and/or postnatal	Perinatal anxiety measure	Perinatal depression measure	Parenting behavior measure	Parenting behaviors/constructs assessed by the study (parenting dimensions)	Main findings association anxiety and depressive symptoms with parenting behaviors/constructs
Aran et al. [55] Australia	Clinical	Longitudinal	5 weeks prenatal: W1: <20 GW; W2:3rd trimester of pregnancy; postnatal: W3: delivery; W4:6 months PP; W5:12 months PP	STAI-T/S	EPDS	EAS	Emotional availability (P)	Association between higher anxiety and depressive symptoms (W1–W4) and lower emotional availability (W4, postnatal). Association between higher comorbidity symptoms and lower emotional availability (W4, postnatal)

Crugnola et al. [56] Italy	Clinical	Cross-sectional	1 week postnatal: 3 months PP	STAI-Y	EPD < S	CARE-Index	Sensitivity (P), controlling (C), and responsiveness (P)	Association between higher postnatal depressive symptoms and higher controlling. Association between higher postnatal anxiety symptoms and lower sensitivity and controlling. No association between depressive symptoms and sensitivity. No association between both depressive and anxiety symptoms responsiveness

Della Vedova et al. [57] Italy	Community	Longitudinal	2 weeks prenatal: W1:3rd trimester of pregnancy; postnatal: W2:3 months PP	STAI-Y	CES-D	CARE-Index	Sensitivity (P), unresponsiveness (P), and controlling (C)	Association between higher levels of depressive symptoms (W1, prenatal) and lower unresponsiveness (W2, postnatal). No associations between prenatal and postnatal state anxiety symptoms, postnatal depressive symptoms and sensitivity, unresponsiveness, and controlling. No associations between prenatal depressive symptoms and sensitivity and controlling

Dib et al. [58] Brazil	Clinical	Cross-sectional	1 week postnatal: 14 months PP	STAI-T/S	BDI-II	DIAP	Sensitivity (P), cognitive stimulation (GL), disengagement (GL), and intrusiveness (GL)	Association between higher anxiety symptoms and greater intrusiveness. No association between anxiety symptoms and sensitivity, cognitive stimulation, and disengagement. No association between depressive symptoms and sensitivity, cognitive stimulation, intrusiveness and disengagement. Association between higher comorbidity with lower sensitivity and cognitive stimulation, and greater disengagement and intrusiveness

Ferber and Feldman [59] Israel	Community	Longitudinal	2 weeks postnatal: W1:2 days PP; W2:6 weeks PP	STAI-T	EPDS	CIB	Sensitivity (P) and intrusiveness (GL)	Association between higher anxiety symptoms (mean score of W1 and W2, postnatal) and lower sensitivity. No association between anxiety symptoms (mean score of W1 and W2, postnatal) and intrusiveness. No association between depressive symptoms (mean score of W1 and W2, postnatal) and sensitivity and intrusiveness

Hakanen et al. [60] Finland	Community	Longitudinal	5 weeks prenatal: W1:14 GW; W2:24 GW; W3:34 GW; postnatal: W4:3 months PP; W5:6 months PP; W6:8 months	SCL-90; PRAQ-R2	EPDS	EAS	Sensitivity (P), structuring (C), intrusiveness (GL), and involvement (P)	Association between higher pregnancy-related anxiety symptoms (W2, prenatal) and intrusiveness (W6, postnatal). Association between higher general anxiety symptoms (W3, prenatal) and intrusiveness (W6, postnatal). Association between higher depressive symptoms (W3, prenatal) and intrusiveness (W6, postnatal). Association between higher depressive symptoms (W4, W5 postnatal) and lower involvement (W6, postnatal). Association between higher depressive symptoms (W5, prenatal) and structuring (W6, postnatal). No association between remaining anxiety and depressive symptoms and parenting behaviors

Hart et al. [61] USA	Community	Cross-sectional	1 week postnatal: 9/16 days PP	STAI-S	EPDS	Breastfeeding behavior	Sensitive positioning (P)	Association between higher depressive symptoms and lower sensitive positioning.No association between anxiety symptoms and sensitive positioning

Holmberg et al. [62] Finland	Community	Longitudinal	9 weeks prenatal: W1:14 GW; W2:24 GW; W3:34 GW; postnatal: W4:3 months PP; W5:6 months PP; W6:8 months PP; W7:12 months PP; W8:24 months PP; W9:30 months PP	SCL-90	EPDS	EAS	Sensitivity (P)	Association between “high and slightly decreasing” depressive symptoms throughout pre-and postnatal periods (W1, prenatal - W5, postnatal) and lower sensitivity (W6, postnatal), compared to the class of “low and stable” depressive symptoms. Association between “high and slightly decreasing” anxiety symptoms throughout pre and postnatal periods (W1, prenatal - W5, postnatal) and lower sensitivity (W6, postnatal), compared to the class of “low and stable” anxiety symptoms

Ierardi et al. [63] Italy	Community	Cross-sectional	1 week postnatal: 3 months PP	STAI-Y	EPDS	CARE-Index	Sensitivity (P), controlling (C), and unresponsiveness (P)	Association between higher depressive symptoms and lower sensitivity. Association between higher state anxiety symptoms with lower sensitivity. No association between anxiety and depressive symptoms and unresponsiveness and controlling

Ierardi et al. [64] Italy	Community	Cross-sectional	1 week postnatal: 3 months PP	STAI-Y	EPDS	CARE-Index	Sensitivity (P), controlling (C), and responsiveness (P)	Association between higher depressive symptoms and lower sensitivity and greater controlling. Association between higher state anxiety symptoms and lower sensitivity and greater controlling No association between anxiety and depressive symptoms and responsiveness

Kaplan et al. [65] USA	Community	Longitudinal	2 weeks prenatal: W1:2nd trimester of pregnancy; postnatal: W2:4 months PP	STAI-T (S)	CES-D	EAS	Sensitivity (P)	No association between depressive and anxiety symptoms and sensitivity

Keren et al. [66] Israel	Clinical	Cross-sectional	1 week postnatal: When the infant's medical condition stabilized - after birth	STAI-T	BDI	CIB	Maternal adaptation (P)	Association between higher postnatal depressive symptoms and higher adaptation. No association between anxiety symptoms and adaptation

Neri et al. [67] Italy	Community	Cross-sectional	1 week postnatal: 3 months PP	PSWQ	EPDS	GRS	Intrusiveness (GL), remoteness (GL), sensitivity (P), and responding to infant cues (P)	Association between higher postnatal depressive symptoms and lower remoteness. Association between higher postnatal anxiety symptoms and lower sensitivity. No association between depressive symptoms and intrusiveness, and sensitivity. No association between anxiety symptoms and intrusiveness and remoteness

Ojo et al. [68] USA	Community	Longitudinal	2 weeks prenatal: W1: during pregnancy; postnatal: W2:4–13 months PP	GAD-7	CES-D	NICHD	Sensitive parenting (P) and harsh parenting (C)	Association between higher depressive symptoms (W2, postnatal) and lower sensitive parenting. No association between depressive symptoms (W2, postnatal) and harsh parenting. Association between higher anxiety symptoms (W2, postnatal) and lower sensitive parenting. No association between anxiety symptoms (W2, postnatal) and harsh parenting

Parfitt et al. [42] UK	Community	Longitudinal	2 weeks prenatal: W1: pregnancy; postnatal: W2:3 months PP	HADS	HADS	CARE-Index	Responsiveness (P), sensitivity (P), and controlling (C)	Association between higher anxiety symptoms (W1, prenatal) and higher controlling and lower responsiveness. No association between anxiety symptoms (W1, prenatal) and sensitivity. No association between postnatal anxiety symptoms responsiveness, controlling, and sensitivity. No association between depressive symptoms (pre or postnatal) and responsiveness, controlling, and sensitivity

Sandre et al. [69] Canada	Community	Cross-Sectional	1 week postnatal: 26–31 weeks PP	IDAS-II	IIDAS-II	PCIRS-IA	Sensitivity (P), intrusiveness (GL) and warmth (P)	Association between higher postnatal depressive symptoms and lower sensitivity and lower warmth. No association between postnatal depressive symptoms and intrusiveness. No association between anxiety symptoms and sensitivity, intrusiveness, and warmth

Stein et al. [70] USA	Community	Longitudinal	3 weeks postnatal: W1:9 weeks PP; W2:3 months PP; W3:6 months PP; W4:10 months PP	GAD-Q	EPDS	Free Play Interaction	Responsiveness (P), control (C), emotional engagement (GL), and vocalizations (P)	Association between higher anxiety symptoms (W1-W2, postnatal) and lower responsiveness (W3). Association between higher depressive symptoms (W1-W2, postnatal) and lower responsiveness. No association between both depressive and anxiety symptoms and control, emotional engagement, and vocalizations

Tluczek et al. [71] USA	Clinical	Cross-sectional	1 week postnatal: W1: home visit between 3 and 19 weeks PP	STAI-S	CES-D	PCERA	Task-oriented behavior (GL), responsiveness (P), and sensitivity (P)	No association between depressive or anxiety symptoms and task-oriented behavior, responsiveness, and sensitivity

Warnock et al. [53] Canada	Community	Longitudinal	2 weeks prenatal: W1:2nd trimester of pregnancy; Postnatal: W2:36 h after birth	HAM-A	EPDS; HAM-D	MBCS	Responsiveness (P)	Association between higher anxiety symptoms (W1, prenatal) and lower responsiveness (W2). Association between higher depressive symptoms (W1, prenatal) and lower responsiveness (W2)

Weiss et al. [54] USA	Community	Longitudinal	2 weeks postnatal: W1:6 months PP; W2:12 months PP	STAI-T/S; GAD-A; anxiety symptoms subscale of EPDS	BDI-II; PHQ-9	PCERA	Positive engagement (GL) and intrusiveness (GL)	Association between higher anxiety symptoms (W1, postnatal) and higher intrusiveness (W2, postnatal). Association between higher depressive symptoms (W1, postnatal) and higher intrusiveness (W1 and W2, postnatal). Association between higher anxiety symptoms (W1, postnatal) and lower positive engagement (W2, postnatal). No association between depressive symptoms and positive engagement

*Note*: C, control dimension; GL, guided learning dimension; P, protection dimension.

Abbreviations: BDI, Beck Depression Inventory; BDI-II, Beck Depression Inventory-II; CES-D, Center for Epidemiologic Studies Depression Scale; CIB, Coding Interactive Behavior; EAS, Emotional Availability Scales; EPDS, Edinburgh Postnatal Depression Scale; GAD, Generalized Anxiety Disorder; GAD-Q, Generalized Anxiety Disorder Questionnaire; GRS, Global Rating Scale; GW, Gestational Week; HADS, Hospital Anxiety and Depression Scale; HAM-A, Hamilton Anxiety Rating Scale; IDAS-II, Inventory of Depression and Anxiety Symptoms; MBCS, Maternal Behavior and Child Self-Regulation; PCERA, Parent-Child Early Relational Assessment; PHQ-9, Patient Health Questionnaire-9; PP, postpartum; PRAQ-R, Pregnancy-Related Anxiety Questionnaire-Revised; PSWQ, Penn State Worry Questionnaire; STAI-T/S, State–Trait Anxiety Inventory-Trait/State; STAI-Y, State–Trait Anxiety Inventory-Y; W, wave.

## Data Availability

Data sharing not applicable–no new data generated.

## Supporting Information

Supporting Information 1 Table S1: presenting the detailed key search strategy used for each one of the databases used to identify relevant studies included in the systematic review.

Supporting Information 2 Table S2: presenting the risk of bias assessment scores for each article included in the systematic review, evaluated using the Critical Appraisal Skills Programme [50]. The table reports individual scores for key sections, with a total quality appraisal score provided for each study, reflecting overall risk of bias.
