# Supplementary material for: Perinatal Anxiety and Depressive Symptoms and Maternal Parenting Behavior During the First Three Years Postpartum: A Systematic Review
Source: Depress Anxiety. 2025 May 19;2025:1801371. doi: 10.1155/da/1801371 (PMC12105896; doi:10.1155/da/1801371)
Supplement: Supporting Information 1 — Table S1: presenting the detailed key search strategy used for each one of the databases used to identify relevant studies included in the systematic review. [file 1801371.f1.docx]

**Supplementary Table 1**

Search terms for each of the databases

| **Search terms** |
| --- |
| 1) Web of Science: anxi* OR depress* OR "mental health" OR psychopathol* (Topic) AND mother* OR maternal* OR paternal* OR parent* OR bab* OR infant* OR child* OR toddler* (Topic) AND interaction (Topic) AND pregnancy OR *natal OR *partum OR gestation (Topic)  2) SCOPUS: TITLE-ABS-KEY ( anxi* OR depress* OR "mental health" OR psychopathol* ) AND TITLE-ABS-KEY ( mother* OR maternal* OR paternal* OR parent* OR bab* OR infant* OR child* OR toddler* ) AND TITLE-ABS-KEY ( interaction ) AND TITLE-ABS-KEY ( pregnancy OR *natal OR *partum OR gestation )  3) PUBMED: (((anxi*[Title/Abstract] OR depress*[Title/Abstract] OR "mental health"[Title/Abstract] OR psychopathol*[Title/Abstract]) AND (mother*[Title/Abstract] OR maternal*[Title/Abstract] OR paternal*[Title/Abstract] OR parent*[Title/Abstract] OR bab*[Title/Abstract] OR infant*[Title/Abstract] OR child*[Title/Abstract] OR toddler*[Title/Abstract])) AND (interaction[Title/Abstract])) AND (pregnancy[Title/Abstract] OR *natal[Title/Abstract] OR *partum[Title/Abstract] OR gestation[Title/Abstract])  4) EBSCO: AB (anxi* OR depress* OR "mental health" OR psychopathol*) AND AB (mother* OR maternal* OR paternal* OR parent* OR bab* OR infant* OR child* OR toddler*) AND AB(interaction) AND AB (pregnancy OR *natal OR *partum OR gestation) |
