# Supplementary material for: Perinatal Anxiety and Depressive Symptoms and Maternal Parenting Behavior During the First Three Years Postpartum: A Systematic Review
Source: Depress Anxiety. 2025 May 19;2025:1801371. doi: 10.1155/da/1801371 (PMC12105896; doi:10.1155/da/1801371)
Supplement: Supporting Information 2 — Table S2: presenting the risk of bias assessment scores for each article included in the systematic review, evaluated using the Critical Appraisal Skills Programme [50]. The table reports individual scores for key sections, with a total quality appraisal score provided for each study, reflecting overall risk of bias. [file 1801371.f2.docx]

**Supplementary Table 2** Risk of bias assessment (*N* = 20)

| Authors. Year | Section A: Are the results of the study valid? | | | | | | | | Section B: What are the results? | | | Section C: Will the results help locally? | | | Quality appraisal (total score) |
| --- | --- | --- | --- | --- | --- | --- | --- | --- | --- | --- | --- | --- | --- | --- | --- |
|  | **Q1** | **Q2** | **Q3** | **Q4** | **Q5a** | **Q5b** | **Q6a** | **Q6b** | **Q7** | **Q8** | **Q9** | **Q10** | **Q11** | **Q12** |  |
| Aran et al., 2021 | 1 | 0.5 | 0.5 | 1 | 1 | 1 | 0.5 | 1 | 1 | 0.5 | 1 | 1 | 1 | 1 | 12 |
| Crugnola et al., 2022 | 1 | 1 | 1 | 1 | 1 | 1 | 0.5 | 1 | 1 | 1 | 1 | 1 | 1 | 1 | 13.5 |
| Della Vedova et al., 2023 | 1 | 1 | 1 | 1 | 1 | 1 | 0.5 | 0.5 | 1 | 1 | 1 | 1 | 1 | 1 | 13 |
| Dib et al., 2019 | 1 | 0 | 1 | 1 | 1 | 1 | 0 | 1 | 1 | 0.5 | 0.5 | 1 | 0.5 | 0.5 | 10 |
| Ferber & Feldman., 2005 | 1 | 1 | 1 | 1 | 0.5 | 0.5 | 0 | 0.5 | 1 | 0.5 | 0.5 | 0.5 | 1 | 0 | 9 |
| Hakanen et al., 2019 | 1 | 1 | 1 | 1 | 1 | 0 | 1 | 1 | 1 | 0.5 | 0.5 | 1 | 1 | 0.5 | 11.5 |
| Hart et al., 2011 | 1 | 0 | 1 | 1 | 0 | 1 | 0 | 0 | 1 | 1 | 1 | 0 | 1 | 1 | 9 |
| Holmberg et al., 2022 | 1 | 1 | 1 | 1 | 1 | 1 | 0 | 1 | 1 | 1 | 1 | 0.5 | 1 | 1 | 12.5 |
| Ierardi et al., 2018 | 1 | 0.5 | 1 | 1 | 1 | 1 | 0 | 0 | 1 | 1 | 1 | 1 | 1 | 0 | 10.5 |
| Ierardi et al., 2022 | 1 | 1 | 1 | 1 | 1 | 1 | 1 | 0 | 1 | 1 | 1 | 0 | 1 | 0.5 | 11.5 |
| Kaplan et al., 2007 | 1 | 1 | 1 | 1 | 1 | 0.5 | 0.5 | 0.5 | 1 | 1 | 1 | 0 | 1 | 0.5 | 11 |
| Keren et al., 2003 | 1 | 0.5 | 1 | 1 | 0 | 1 | 0 | 0.5 | 1 | 0.5 | 1 | 0.5 | 1 | 1 | 10 |
| Neri et al.,2015 | 1 | 1 | 1 | 1 | 1 | 1 | 0.5 | 1 | 1 | 0 | 0.5 | 1 | 1 | 0.5 | 11.5 |
| Ojo et al., 2021 | 1 | 0.5 | 1 | 1 | 1 | 1 | 1 | 1 | 1 | 1 | 1 | 0.5 | 1 | 1 | 13 |
| Parfitt et al., 2013 | 1 | 1 | 1 | 1 | 0 | 0 | 0.5 | 0.5 | 1 | 1 | 1 | 0.5 | 1 | 1 | 10.5 |
| Sandre et al., 2022 | 1 | 1 | 1 | 1 | 1 | 1 | 1 | 0.5 | 1 | 1 | 1 | 1 | 1 | 1 | 13.5 |
| Stein et al. , 2012 | 1 | 1 | 1 | 1 | 1 | 1 | 1 | 1 | 1 | 1 | 1 | 1 | 1 | 1 | 14 |
| Tluczek et al., 2010 | 1 | 0 | 1 | 1 | 1 | 1 | 0.5 | 0 | 1 | 1 | 1 | 0.5 | 1 | 1 | 11 |
| Warnock et al., 2016 | 1 | 1 | 1 | 1 | 1 | 1 | 0 | 0 | 1 | 0.5 | 0.5 | 0 | 1 | 0.5 | 9.5 |
| Weiss et al., 2023 | 1 | 1 | 1 | 0.5 | 1 | 1 | 0.5 | 1 | 1 | 0 | 1 | 1 | 1 | 1 | 12 |
| *Note*. Q1: Did the study address a clearly focused issue?; Q2: Was the cohort recruited in an acceptable way?; Q3: Was the exposure accurately measured to minimise bias?; Q4: Was the outcome accurately measured to minimise bias?; Q5a: Have the authors identified all important confounding factors?; Q5b: Have they taken account of the confounding factors in the design and/or analysis?; Q6a: Was the follow up of subjects complete enough?; Q6b: Was the follow up of subjects long enough?; Q7: What are the results of this study?; Q8: How precise are the results?; Q9: Do you believe the results?; Q10: Can the results be applied to the local population?; Q11: Do the results of this study fit with other available evidence?; Q12: What are the implications of this study for practice? | | | | | | | | | | | | | | | |
